# Tau and DNA Damage in Neurodegeneration

**DOI:** 10.3390/brainsci10120946

**Published:** 2020-12-07

**Authors:** Luca Colnaghi, Diego Rondelli, Marco Muzi-Falconi, Sarah Sertic

**Affiliations:** 1 Department of Molecular Biochemistry and Pharmacology, Istituto di Ricerche Farmacologiche Mario Negri IRCCS, Via Negri 2, 20156 Milan, Italy; 2 Dipartimento di Bioscienze, Università degli Studi di Milano, Via Celoria 26, 20133 Milan, Italy; diego.rondelli@unimi.it (D.R.); marco.muzifalconi@unimi.it (M.M.-F.)

**Keywords:** tau, DNA damage, Alzheimer’s disease

## Abstract

Neurodegenerative disorders are a family of incurable conditions. Among them, Alzheimer’s disease and tauopathies are the most common. Pathological features of these two disorders are synaptic loss, neuronal cell death and increased DNA damage. A key pathological protein for the onset and progression of the conditions is the protein tau, a microtubule-binding protein highly expressed in neurons and encoded by the *MAPT* (microtubule-associated protein tau) gene. Tau is predominantly a cytosolic protein that interacts with numerous other proteins and molecules. Recent findings, however, have highlighted new and unexpected roles for tau in the nucleus of neuronal cells. This review summarizes the functions of tau in the metabolism of DNA, describing them in the context of the disorders.

## 1. Introduction

Neurodegeneration is the gradual degeneration and death of neurons in the central or peripheral nervous systems [1]. Alzheimer’s disease (AD) is among the most common neurodegenerative diseases, and this debilitating and incurable condition is rapidly becoming even more common with the aging of the population [2]. Although the cause of AD is still not clear, its onset and progression correlate with levels of hyperphosphorylated and aggregated tau [3,4,5,6,7,8,9].

Tau is a microtubule-associated protein (MAP) encoded by the *MAPT* gene [10]. In healthy adult subjects, tau is a soluble, non-hyperphosphorylated protein that can promote the self-association of tubulin into microtubules [11,12,13]. Humans express six tau isoforms, all derived from alternative mRNA splicing of the *MAPT* gene. In neurons, all isoforms are mainly localized in axons; they are natively unfolded and can bind and interact with the microtubule [11,14,15,16,17]. Tau isoforms interact with other proteins and with nucleic acids besides the microtubule [18,19].

In AD, tau is abnormally phosphorylated by numerous kinases at several sites [20]. Hyperphosphorylated tau is the principal component of neurofibrillary tangles (NFTs), a pathological hallmark of AD that correlates with severity of dementia [21,22]. Although the role of the hyperphosphorylation of tau is debated [23], a leading hypothesis suggests that the aberrant phosphorylation is required to induce aggregation of the protein to form tau oligomers and NFTs [23]. While NFTs can be formed by wild-type tau [24], tau mutants often have a reduced ability to bind the microtubule and a higher propensity to be insoluble and form NFTs [25,26]. Tau NFTs are also found in other neurodegenerative disorders, such as frontotemporal dementia, progressive supranuclear palsy, corticobasal degeneration and Pick’s disease, collectively called tauopathies [27,28]. While tau pathology is at the base of all these conditions, the structures of tau aggregates and filaments that can be found in these disorders vary [6,7,8,9]. Overall, this evidence suggests a predominant role of tau hyperphosphorylation and aggregation in the onset and progression of AD and a promising pharmaceutical target [29,30]. A second pathological marker of AD are extracellular amyloid-β plaques (Aβ). Their role in AD is still debated, since their presence does not correlate with AD onset and progression [31]. However, some species of Aβ oligomers are toxic to neurons, can induce neurodegeneration and may be a valid therapeutic target for AD [31,32,33].

Several types of post-translational modification have been described for tau, phosphorylation being the most studied [34]. This is because abnormally and hyperphosphorylated tau has been found in tau NFTs purified from AD patients. Although tau phosphorylation and deposition correlate with neurodegeneration, the mechanisms responsible for tau toxicity are still unclear [35,36]. This has led to therapies with limited efficacy and tauopathies are currently untreatable.

In the past decade, new and unexpected roles of tau have emerged in the nucleus and in the maintenance of genome stability [19,37]. These findings are supported by the fact that DNA lesions are recurrent in post-mortem brains of subjects with neurodegenerative diseases [38,39].

Maintaining the integrity of the genome is crucial for the physiology of any nucleated cell [40]. Our genome can, however, be attacked by endogenous and exogenous sources that can induce DNA damage. If not properly repaired, DNA lesions may cause mutations, altering cellular processes or leading to cell death [41]. Research on DNA repair mechanisms has mainly focused on cycling cells, in view of the high incidence of DNA mutations and chromosomal rearrangements in proliferating cancer cells [42]. These studies have shown that mammalian cells have developed several specific pathways to recognize and repair DNA lesions and collectively they constitute the DNA damage response (DDR) [43]. The importance of a functional DDR is illustrated by the many cancer susceptibility disorders caused by DNA repair deficiencies such as ataxia–telangiectasia, xeroderma pigmentosum, Nijmegen breakage syndrome, Bloom syndrome, Werner syndrome and many others [44,45,46].

**Figure 1:**
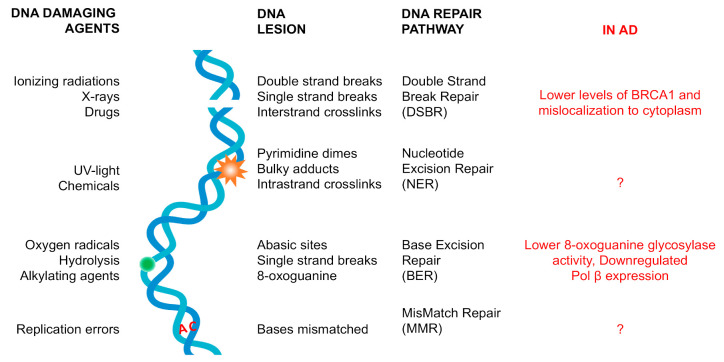
Types of DNA lesions, their DNA repair pathways and possible connections with Alzheimer’s Disease (AD). The principle and well-characterized DNA damage repair pathways are double-strand break repair (DSBR), nucleotide excision repair (NER), base excision repair (BER) and mismatch repair (MMR). DSBR- and BER-specific mutations have been associated with AD (red).

The nature of DNA damage that the genome can develop varies widely and to face this variety, cells utilize several repair mechanisms, each specialized in a different type of DNA lesion. In mammals, at least five major DNA repair pathways safeguard the genome (Figure 1) and they not only act on the metabolism of DNA, but also modulate cell cycle checkpoint proteins to affect cell cycle progression [47]. The major repair pathways are base excision repair (BER), mismatch repair (MMR), nucleotide excision repair (NER) and DNA double-strand break (DSB) repair, mediated by homologous recombination (HR) and/or nonhomologous end joining (NHEJ) [47]. BER is the main pathway for the repair of damaged DNA bases that do not significantly distort the structure of the DNA helix, such as oxidized nucleotides [48]. There are two general BER pathways: short-patch and long-patch BER. The former leads to repair of a tract of a single nucleotide and the latter to a tract of at least two nucleotides. The MMR pathway is mainly involved in the repair of misincorporated nucleotides that have been inserted by the replicative polymerases [49]. NER can recognize and remove different types of bulky and helix-distorting lesions from DNA. There are two NER pathways: transcription coupled-NER, which removes bulky lesions formed on transcribed sequences, and global genome-NER to remove distorting lesions in the whole genome [50]. HR and NHEJ are specialized pathways that repair DNA DSBs [43].

DSBs are considered to be amongst the most dangerous types of DNA damage. In mammals, a single unrepaired DSB can lead to cell death [51], and errors in their processing may cause deletions or chromosomal aberrations, which, in turn, can trigger the development of cancer or other genomic instability disorders [52]. Thus, DSB repair is critical for cell survival and the maintenance of genomic integrity. HR [53] and NHEJ [54] differ in their requirement for a homologous template DNA and the fidelity of DSB repair. HR repairs lesions in an error-free fashion, since it uses the undamaged sister chromatid as a template. It is therefore restricted to the late S and G2 phases of the cell cycle [52]. NHEJ, on the other hand, is more error-prone, and repairs DSBs by direct ligation of the broken ends, with minimal processing. NHEJ is believed to be active in all phases of the cell cycle [54].

While all these DDR pathways are functional in proliferating cells, profound differences in DNA repair mechanisms have been reported for postmitotic cells such as neurons [55,56,57]. The onset and role of DNA lesions in AD and their connection with tau pathology are not clear (Figure 1, red column). The aim of this review is therefore to describe the latest findings that may link tau and/or pathological tau to DNA lesions and neurodegenerative processes in tauopathies.

## 2. Neurons and the Repair of DNA Lesions

Neurons are the longest-living postmitotic cells in the human body [58]. To function for decades, they have to safeguard their genomes from endogenous and, to a lesser extent, exogenous DNA insults [55]. The main threat to their genomes comes from endogenous sources in the forms of (i) cytotoxic reactive oxygen species (ROS) that can cause oxidative DNA damage, and (ii) activity-dependent transcription that induces DSBs [59,60]. Exogenous sources are curbed by the blood brain barrier (BBB), which spares brain cells from the majority of the exogenous DNA insults, limiting, therefore, the possible sources of DNA lesions [61]. Nevertheless, a handful of chemicals, such as the common carcinogen benzo[a]pyrene, can pass the BBB and cause DNA lesions [62].

Oxidized DNA is believed to be the most common DNA lesion in neurons. This is unavoidable due to their high metabolic activity [63]. The BER pathway is therefore probably the most active DDR mechanism in neuronal cells. Similarly, to cycling cells, in neurons, the oxidized nucleotide is recognized and removed by a DNA glycosylase that starts BER and generates an abasic site. The lesion is further processed by short-patch or long-patch repair to complete BER [48].

Aside from oxidized DNA, a second major threat to neuronal genomes comes from DNA DSBs arising from the development and plasticity of the brain. Neuronal activity triggers the expression of immediate early genes, usually transcription factors, which mediate neuronal physiology by regulating the expression of several downstream targets. To ensure fast activation of immediate early genes, neurons resolve DNA topological constraints in their promoters by breaking the double helix. The creation and repair of the resulting DNA DSBs are apparently mediated by topoisomerase IIβ (Topo IIβ) and can be visualized by the detection of histone γH2AX foci, a general marker of DNA DSBs [60,64]. However, inhibition of the DNA-dependent protein kinase (DNA-PK), a required component in the activation of NHEJ [65,66,67], prevents the repair of activity-induced DSBs, thus suggesting a more complex mechanism of repair of these breaks, where the role of NHEJ is still unclear [64]. Sleep, a state characterized by a decline in neuronal firing, has been proposed as required to reduce the number of DSBs in single neurons [68,69]. This suggests somehow that DNA repair is facilitated in resting state neurons.

Initial interest in neuronal DNA repair however did not stem from physiological processes but from the neurological defects observed in several inherited conditions, where mutations in DNA repair genes caused premature neuronal death. Examples are conditions such as xeroderma pigmentosum (an autosomal recessive genetic disorder in which NER is aberrant), Cockayne’s Syndrome (another condition belonging to the NER-related family of disorders) and ataxia–telangiectasia syndrome (with mutations in the Ataxia Telangiectasia Mutated (ATM) apical kinase) [70]. There is thus overwhelming evidence that genome maintenance is as important in neurons as in all other tissue cells. In addition, like in other somatic cells, DNA repair in these highly specialized cells is not without mistakes. Unfaithful DNA repair has been confirmed by recent single-cell whole-genome sequencing, proposing that with time, neurons accumulate somatic mutations [71,72]. This suggests that genomic instability in neurons may be a part of normal aging. Whether this is also at the basis of neurological disorders, and whether tau has any role in this, is still not clear.

## 3. Neurodegeneration and DNA Lesions

The pathophysiology of tauopathies, including AD, consists of synaptopathy, changes in neurotransmitter expression, accumulation of intracellular tau NFTs, neuronal loss and brain atrophy [2,73]. Accumulation of unrepaired DNA is another feature of AD [74], while for other tauopathies it has been only briefly explored [38,39,75,76,77,78]. The lesions are likely caused by ROS produced by Aβ and tau oligomers, and their onset is accompanied by the activation of DDR kinases, such as ATM, that can phosphorylate a plethora of targets [79,80].

Compared to age-matched controls, histone γH2AX-positive neurons are increased in the hippocampus and frontal cortex of subjects suffering from mild cognitive impairment or AD [74,81]. This suggests there may be more DSBs in the genome of those neurons. Mild cognitive impairment is a preclinical stage of AD, and the presence of DNA DSBs can suggest a role of DNA lesions in the early stages [82]. DNA DSBs are not, however, the only DNA lesions described in the literature. DNA single strand breaks (SSBs) are also reported in the hippocampi of AD brains [39]. Among the likely causes of SSBs and DSBs, the majority of DNA lesions in neurons are caused by ROS. Nuclear and mitochondrial DNA derived from brain cells of subjects with reported neurodegenerative diseases contain more oxidative DNA lesions, detected by 8-hydroxy-2′-deoxyguanosine (8-OHdG), a widely used marker of DNA oxidation [83,84,85]. However, it is still debated why these DNA lesions accumulate in AD and cause DNA SSBs and DSBs, but it could in part be due to a reduction in their repair. 8-Oxoguanine glycosylase activity is lower in AD than controls [86] and the expression of POLβ, the main DNA polymerase involved in BER, is also downregulated (Figure 1, red). This can also be seen in mild cognitively impaired subjects [86].

Along with proteins belonging to BER, other DDR genes are reported to be affected in AD. ATM is reduced in the frontal cortex of AD brains and also downregulated in mouse models of AD [80]. Evidence from human AD samples and animal models suggests that NHEJ is affected in AD pathology [87,88].

DNA DSBs formation may also depend on the physiological activity of neurons in the pathological context of AD [60]. Following activity, DNA DSBs were increased by exposure to amyloid-β oligomers [60]. This increase may be linked to the ability of Aβ to lower the levels of the DNA repair protein BRCA1 and the activity of DNA-PK [89,90]. All this suggests a direct link between neuronal activity, DNA lesions and a pathological marker of AD. Tau ablation reduced Aβ effects on DSBs in neurons [60], but the role of tau oligomers in activity-dependent DNA DSBs in AD has not been explored.

BRCA1, a critical regulator of DNA repair, cell cycle checkpoint control and maintenance of genomic stability, may also have a role in AD. In neurons, its protein levels are regulated by neuronal activity and seem to be low in the brains of AD patients [90,91]. In vitro, Aβ oligomers reduced the levels of BRCA1 in primary neuronal cultures [90]. Apart from its expression, the localization of BRCA1 also seems to be affected by AD. In neurons from young familial AD subjects, BRCA1 shows cytosolic localization and phosphorylation after DDR activation [92]. In the cytosol, BRCA1 co-localizes with presenilin 1 (PS1) [91], a component of a multi-subunit protease that targets the amyloid precursor protein (APP) to make Aβ. Mutations in the gene encoding PS1 are the most common cause of familial AD [93], thus strongly linking BRCA1 with the pathology. BRCA1 may also have a role in sporadic AD. The protein was phosphorylated in post-mortem brains of sporadic AD patients positive to Aβ plaques [94].

## 4. Neurodegeneration, Cell Cycle Regulation and DNA Damage

Neurons, once they differentiate, cannot proliferate [58]. This seems to be due to cell cycle suppression, an active process that restrains cell cycle progression. In vivo and in vitro data indicate that various insults can loosen these restraints, pushing neuronal cells to re-enter the cell cycle, and cell death. Originally it was thought that when cell cycle progression restarts, neurons reach the G1/S boundary and cell death occurs by apoptosis before new DNA synthesis [95]: a process regulated by CDKs (Cyclin Dependent Kinase), transcription factors and effectors of the DNA damage checkpoint such as ATM and the tumor suppressor p53 [96]. However, there are exceptions. During development, for instance, some neurons re-enter the cell cycle, replicate their DNA and reach the G2/M transition as tetraploid cells where they stop proliferating; mitosis never takes place and these remain viable [97,98]. The reason why some neurons become polyploid is currently debated. In healthy neurons, re-entry into cell cycle progression and passing the G2/M boundary is very rare, and usually leads to the death of these neurons [58,99].

A connection between neurodegeneration and cell cycle re-entry was proposed when phosphorylated forms of tau, normally found in cycling cells, were identified in neurons from AD patients. This led to discovering that the percentage of neurons that reactivate their cell cycle is doubled in AD. Neuronal DNA replication has been extensively documented in AD, suggesting progression past the G1/S boundary [77,100]. More likely, therefore, in AD, neurons can reach the G1/S boundary, replicate their DNA and successfully enter G2 as tetraploid neurons. Mitosis, however, is very rare, and neurons block their cell cycle at the G2/M transition and can survive for years [96,101,102,103,104]. The reason for this is also still not clear. A leading hypothesis suggests, however, that DNA lesions and their repair may have a role. During neurodegenerative processes, unrepaired DNA lesions may push the cells to re-enter cell cycle progression [105,106]. This evidence comes from i) the description of cell cycle restarts in early neurodegenerative processes [107,108] and ii) AD patient specimens that have higher levels of cell cycle protein expression than healthy controls [100]. The presence of tetraploid neurons in the cerebral cortex is an early sign of AD [101]. This is seen in the absence of tau NFT, suggesting that tetraploidy may precede the neuropathological signs of AD [109]. How, in AD, neurons reactivate their cell cycle and replicate their DNA and whether tau has any role in this is still debated, but it is worth noting that cyclin-dependent kinase inhibitors are neuroprotective [110].

## 5. Tau and DNA Lesions

Although tau is primarily considered a cytosolic protein, it is reported to interact with nucleic acids and to localize to the nucleus of neuronal and non-neuronal cells [37,111,112,113,114]. In neurons, tau in the nucleus is hypophosphorylated [19], evidence supported also by studies in neuroblastoma SH-SY5Y cells [115]. In vitro, tau binds the minor groove of the DNA double helix and interacts with single-stranded DNA with less affinity [116,117]. Hyperphosphorylation of tau reduces its ability to bind DNA [118]. Purified tau has higher affinity for DNA than microtubules [119]. The DNA–tau complex shares some structural similarities with the DNA–histones complex and may efficiently preserve DNA [117,120,121]. For instance, the binding protects DNA from ROS, such as hydroxyl radicals, and also raises the melting temperature of the double helix [116,117,122]. The interaction between tau and DNA can also affect tau’s biochemical properties, facilitating its aberrant aggregation. DNA, being a polyanionic molecule, may trigger protein misfolding and insolubility [123]. Interestingly, the DNA of bacteria may be more prone to this than the DNA of eukaryotic cells [124], somehow supporting a positive association between bacterial infection and AD [125,126]. Whether viral DNA has any effects on tau aggregation is still not clear. Nevertheless, viral infection can relocate tau to the nucleus [127].

**Figure 2:**
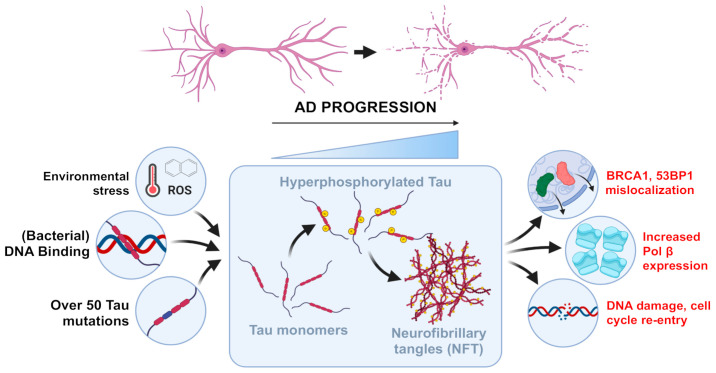
Tau, interaction with DNA damage and AD progression. DNA insults, such as caused by reactive oxygen species (ROS), bacterial infections and tau mutations, can cause hyperphosphorylation of tau and the formation of neurofibrillary tangles (NFT) in AD-induced progression. This particular situation drastically negatively influences DNA damages and repair pathways (Red).

In cultured murine primary neurons, tau binds chromatin in physiological conditions, showing a preference for Adenine and Guanine (AG_-rich regions. This interaction is dynamic and can be modulated under stress conditions [128]. In cells, tau quite likely participates directly in DDR in response to DNA lesions. In the absence of tau, cells have increased phosphorylation of histone H2AX on serine 139 (γH2AX) [129], suggesting more DNA lesions or a longer DNA repair time. Although the mechanism is not fully understood, the main evidence to suggest that tau regulates genomic stability in neurons comes from primary cultures of tau knock-out neurons subjected to heat shock. Hyperthermic conditions trigger the production of ROS that, in turn, can damage the DNA [130]. Compared to wild-type neurons, the cellular stress induces DNA breaks that can be prevented by expression of human tau targeted to the nucleus [19]. This protective mechanism is likely also present in vivo. In the hippocampus and cortex of tau knock-out mice, hyperthermia induces DNA DSBs and histone H2AX phosphorylation [129]. Tau deletion also caused a slower repair of DNA DSBs in the hippocampus of the animals [129], thus suggesting both a DNA protective role and a direct or indirect DNA repair role of tau in the brain. Other than by binding to the DNA, tau can also safeguard the genome indirectly. It can bind the mitotic spindle microtubules, probably similarly to its interaction with the cytoskeleton, to stabilize it [131]. An unstable mitotic spindle can cause chromosomes to mis-segregate and can lead to chromosomal instability [132]. Along this line, peripheral cells of patients with mutated tau present chromosomal aberrations [131,133]. This is further supported by (i) an increase in chromosomal aberrations in tau knock-out mice and (ii) a higher risk of cancers, besides tauopathies, in families carrying tau mutations [133,134]. Tau deletion also affects nuclear organization by affecting heterochromatin and DNA methylation in cultured neurons. The absence of tau causes a surge of DNA breaks in these regions, a finding confirmed in AD patient-derived samples [135,136]. This evidence suggests that (i) tau can maintain genomic stability and that (ii) DNA lesions in AD and tau may be linked (Figure 2). In non-neuronal cells, tau has been proposed to directly affect DNA repair by interacting with p53, Pin1 and PARN by modulating nuclear de-adenylation following UV-induced DNA damage. Further experiments are needed to determine whether this mechanism is present also in neurons and whether it may be relevant to AD [137].

DNA lesions and acute oxidative stress can affect tau directly too. They change its phosphorylation state and also affect its localization. For instance, in SH-SY5Y cells, the DNA-damaging drug etoposide induces the translocation of tau to the nucleus while lowering its phosphorylation levels [115]. Although DNA damage may reduce tau phosphorylation, DDR checkpoint kinases can directly target tau. For instance, checkpoint kinase 2 (Chk2)-dependent phosphorylation of tau enhanced its toxicity in a transgenic *Drosophila* model [138]. Other DDR-related proteins can affect tau phosphorylation. p53 is upregulated in AD brains compared to controls and indirectly, not being a kinase, it induces phosphorylation of tau in HEK293 cells [139,140]. Although the exact function of this link is still debated, it does suggest an undefined role for tau in the cell cycle regulation of neurons undergoing neurodegenerative processes. Overall, however, how pathogenic, hyperphosphorylated and aggregated tau affects DNA lesions and their repair is not fully understood.

As reported above, tau mutations have been associated with a greater predisposition to cancer due to genomic instability [133], but a direct link with an increase in genomic instability in neurons and AD is still missing. Nevertheless, tau’s interaction with DNA may be affected by both aggregation and phosphorylation [141,142], reducing its putative protective function.

In addition, DNA repair too may be altered by tau oligomers [143]. In the transgenic mouse tau model Thy-Tau22, when hippocampal neurons are enriched in tau oligomers at six months of age, Polβ expression is elevated [144]. This is likely to be due to the increase in oxidative stress underlying the onset and progression of the disorder, and not directly to tau oligomers. More directly, however, tau oligomerization is reported to affect the localization of DDR proteins. Tau aggregation mislocalizes BRCA1 and p53-binding protein (53BP1) [94,145] in the cytoplasm of neurons during AD and other tauopathies such as Pick’s disease and progressive supranuclear palsy, causing its insolubility. Sequestration of BRCA1 and 53BP1 in the cytoplasm by hyperphosphorylated and aggregated tau may therefore contribute to the progression of tauopathies [94,145].

## 6. Conclusions

The connection between tau and maintenance of genome integrity has been described by several laboratories. Brain samples from AD patients exhibit neuronal death and accumulation of genomic lesions. Reports suggest that hyperphosphorylated and aggregated tau may impair DNA repair by interacting with, and sequestering to the cytoplasm, DNA repair proteins such as BRCA1. A clear link between tau, DNA damage and neurodegeneration, however, is still missing. Future experiments should investigate the role of tau hyperphosphorylation, tau aggregation and tau mutations in the formation and repair of DNA lesions described in AD neurons. DNA damage and repair pathways have been extensively studied in oncology [146], where DNA mutations are a driving force in cancer growth [40], and several drugs have been developed to target them [147]. A better understanding of the role of DNA lesions in AD could also lead to better and innovative therapeutic regimens. Besides AD, DNA lesions in other tauopathies have received less attention. Finally, loss of genomic integrity might be a common theme in a number of other neurodegenerative disorders: several proteins with the tendency to aggregate aberrantly and cause neuronal death also interact with DNA [148,149]. Examples are Parkinson’s disease, polyglutamine diseases such as Huntington’s disease, and amyotrophic lateral sclerosis [150,151], suggesting, therefore, that DNA lesions and their repair may contribute to neurodegenerative processes.
